# Literacy and attitude of Asian youths on dengue and its prevention in an endemic developed community

**DOI:** 10.3389/fpubh.2024.1361717

**Published:** 2024-03-08

**Authors:** Wern Fern Soo, Kalaipriya Gunasekaran, Ding Xuan Ng, Kylie Kwek, Ngiap Chuan Tan

**Affiliations:** ^1^SingHealth Polyclinics, Singapore, Singapore; ^2^NUS Yong Loo Lin School of Medicine, Singapore, Singapore; ^3^SingHealth Duke-NUS Family Medicine Academic Clinical Program, Singapore, Singapore

**Keywords:** dengue, vector control, youth, knowledge, attitude

## Abstract

**Background:**

Over the past few decades, the incidence of dengue fever has considerably increased. Effective vector control strategies and specific protection using dengue vaccine are thought to be the key elements to combat dengue. The dengue incidence among the Singapore youths (15–24 years) was second only to that of adults (25–44 years). This study evaluated the knowledge and attitude of Singapore youths on dengue and its preventive measures.

**Methods:**

A cross-sectional study using online-based questionnaire survey was conducted among Singapore youths from September to November 2022. Data were analyzed for descriptive statistics whereas Chi-squared test, linear regression analysis and Pearson correlation were used to determine the association between demographic factors and youth’s attitude on dengue prevention using Rstudio.

**Results:**

A total of 624 respondents completed the survey out of 1822 surveys distributed nation-wide, with a response rate of 34.2% (mean age 17.4 years ± 1.84; 59.3% female; 89.9% Chinese). The mean dengue knowledge scores of participants were 14.1 ±2.8. Univariate analysis showed that teenagers (15–19 years) had significantly higher knowledge score than the young adults (20–24 years) (β=0.82,95%CI = 0.13–1.51, *p* = 0.021). Majority of them were aware of the Mozzie Wipeout campaign (90.2%) followed by the release of Wolbachia mosquitos (69.1%). Two-thirds of the youths who were aware of Wolbachia and Gravitrap considered that it was effective in reducing dengue infection rates. Participants suggested information about the current dengue infection rate (71.9%) as the most effective of the five proposed strategies to improve uptake of dengue preventive measures. In comparison to young adults, teenagers were more likely to uptake dengue preventive measures if widespread mosquito control practices were implemented (69.1% vs. 42.3%).

**Conclusion:**

The overall knowledge of the youths on dengue and its prevention was satisfactory. Future health promotion campaigns targeting the youths should focus on transforming the knowledge into practice.

## 1 Introduction

Dengue virus infections, transmitted by *Aedes aegypti* mosquito, is endemic in more than 100 countries from Africa and America to Asia, with Asia representing around 70% of the global disease burden. Most dengue infection is mild in nature, but it can potentially be severe and fatal. With an estimated 100–400 million infections annually, dengue is now a threat to about half of the world population. The dengue incidence has risen significantly over the past few decades from 0.5 million cases in 2,000 to 5.2 million in 2019, while the real number of cases is likely underreported (1). In 2020, Singapore recorded a total of 35,315 dengue infections, almost double the number from 2019 with an IR (incidence rate) of 621 per 100,000 population (2). Dengue infection will continue to rise dramatically worldwide due to climate change and global warming, which will escalate the spread of dengue in endemic areas, with extension to new areas such as Europe (3, 4).

With no specific treatment available, prevention becomes the most effective strategy to curb the spread of the disease. Vector control is crucial due to the unique characteristics of the Aedes mosquitoes and the complex nature of dengue transmission. Vector control strategies encompass a range of approaches that aim to disrupt the mosquito lifecycle, reducing their population and consequently the risk of dengue transmission.

The National Environment Agency (NEA) of Singapore is a government agency overseeing Singapore’s public health standard and ensuring a clean and sustainable environment by working with local community partners and the public. For dengue control, the agency provides updates on the current reported dengue infection rate and areas of dengue clusters under its website and myENV digital app (5). NEA also has launched various vector control measures like “Mozzie Wipeout” campaign, Gravitrap and the project *Wolbachia* (6). The “Mozzie Wipeout” campaign was launched in 2016 to promote source reduction through implementing the 5-step Mozzie Wipeout technique (7). The 5 step Mozzie Wipeout technique consists of Breaking up hardened soil, Lifting and emptying flowerpot plates, Overturning pails and wiping their rims, Changing water in vases and Keeping roof gutters clear and placing BTI (*Bacillus Thuringiensis* Israelensis) insecticide (B-L-O-C-K).

Since 2017, NEA has deployed in-house–developed Gravitraps at various locations throughout Singapore with the intention to trap gravid female *Aedes* on the sticky lining. The traps are monitored every 2 weeks, and Gravitrap indices are mapped on a geographical information system (GIS) to identify areas to intensify source-reduction programs. High Gravitrap index areas are also listed on the NEA website to encourage the local community to undertake appropriate measures to prevent the spread of dengue (6).

Another method using *Wolbachia,* a maternally inherited endosymbiotic intracellular bacterium, suppresses mosquito population when artificially introduced into male *Aedes aegypti*. Male *Wolbachia*-*Aedes* mosquitoes are let to mate with female mosquitoes, yet their resultant eggs do not hatch, and no offspring is produced (6). Despite continued effort in implementing these vector control measures in Singapore, dengue infections were estimated to have reached their second highest peak (32,173) in 2022 (8).

Digital games have been widely used as a medium for learning especially in recent years. “Serious games” are digital games that are built for the purpose of learning and education beyond just entertainment. Through these serious games, participants will be able to learn about the disease and its prevention, treatment and vaccination in an interactive and engaging way which will lead to the intended behavior change. As a significant proportion of the local youths own personal smart phone, there is potential for the use of phone-based games in disseminating important health and disease preventive information to the public.

Community engagement plays an integral role in successful vector control, in which youths (aged 15–24) play a crucial role because of their ability to adopt new behaviors (9). By involving the youths, communities can tap into a subset of the population to complement national efforts in dengue prevention. With IR of 344.9 and 140.9 per 100,000 population (DENV-1 and DENV-2 respectively), dengue incidence among youths was also the second highest after that of adults (25–44 years) among Singapore residents (10). Youths can play a significant role in dengue vector control as they are not only at increased risk of infection but also capable of implementing preventive measures. However, little is known about their level of awareness on dengue disease and its prevention. Hence, this cross-sectional study aims to evaluate the knowledge on dengue disease, level of awareness of its prevention, and the perceived effectiveness of the current measures in vector control among local multi-ethnic Asia youths.

## 2 Materials and methods

### 2.1 Study design, setting, and participants

A cross-sectional, nation-wide, online questionnaire-based survey was co-created by investigators in the Research Department in SingHealth Polyclinics and students in a local center of higher learning. The questionnaire was disseminated to secondary and tertiary educational institutions across Singapore. Convenience sampling technique was employed to recruit participants over 10 weeks from September to November 2022. Inclusion criteria were (1) individuals aged 15–24 years old, (2) have internet access to the questionnaire, regardless of gender or ethnicity.

### 2.2 Sample size estimation

According to Amanah et al. (11), the proportion of undergraduate students with moderate level of knowledge was 62%. Using 99% confidence level and 5% precision, the minimum sample size required is 625 using sample size for proportion. With an estimated average response rate of 35% for internet survey, the survey should be sent out to at least 1786 youths (12, 13).

### 2.3 Implementation of survey

The questionnaire was administered using FormSG, a secure platform for personal data, validated and used by most government agencies and public healthcare institutions and disseminated through social media (14). The questionnaire was standardized such that all participants were asked the same questions in an identical format and responses were recorded in a uniform manner to increase its reliability (15). The purpose and confidentiality was explained prior to the initiation of survey and participation implied consent.

To minimize selection bias by convenience sampling, the link to the questionnaire was posted in public groups, namely WhatsApp™ chat groups and Telegram™ channels (16) dedicated to secondary and tertiary institutions, rather within the personal contacts. These groups are not categorized by specific profession or gender, therefore reducing reliance on the use of referrals, and the findings would be generalizable (17).

### 2.4 Questionnaire design

A total of 43 questions on sociodemographics, awareness of dengue and vector control measures made up the survey. The sociodemographics section examines participant demographics including age, gender and ethnicity. Participants’ knowledge on dengue disease and dengue vaccination was assessed using a composite of agreement scores to five statements.

A set of dengue preventive measures were assembled to assess the participant’s awareness and support of four campaigns using a Likert scale of strongly agree to strongly disagree. Ways to increase participants’ receptiveness to uptake dengue preventive measures was investigated with participants’ selection of one or multiple options of the five proposed strategies. The five strategies to increase the dengue preventive measures uptake are “Home and workplace inspections,” “Information about the current Dengue infection rates,” “Information on effectiveness of preventive measures,” “Widespread mosquito control practices” and “Frequent reminders from official sources.”

### 2.5 Pilot testing of the study tool

User Acceptance Testing (UAT) was conducted in September 2022 with participants of the target age group to identify their difficulties in filling up the e-questionnaire, revise the questions to enhance their clarity and address their doubts before rolling it out to the target population.

### 2.6 Data management

All responses was collected in a .pst file and converted to .csv format using the data collation tool provided by FORMSG. A data analyst ensured that participants completed the survey and met the eligibility criteria for inclusion in analysis. Consequently, the data was stored in a secure password-protected database and assessed only by study team members. Research data used in publication will be kept for a minimum of 10 years before being discarded.

### 2.7 Study outcomes

To understand the overall awareness of youths on dengue and dengue vaccination awareness, five dengue awareness questions are being scored from a scale of 0–4: 0 (Strongly Disagree), 1 (Disagree), 2 (Neutral), 3 (Agree), 4 (Strongly Agree). The five questions are “A person can be infected with Dengue more than once,” “Dengue infection can be fatal,” “Dengue vaccine is available to local people,” “Dengue vaccine can be administered for selected people aged 12–45 years” and “Dengue vaccine is effective in reducing the infection.” Subsequently, the scores for these questions are summed up and a score ranging from 0 to 20 will be obtained. A higher score indicates higher awareness on dengue and dengue vaccination.

For questions relating to the effectiveness of vector control measures, participants that responded with “Strongly Agree” or “Agree” were categorized as “Agree,” while “Strongly Disagree” or “Disagree” were grouped as “Disagree.” These questions were focused on participant’s perceived effectiveness of prevention measures, such as Wolbachia mosquitoes, Gravitrap, fumigation and serious games.

### 2.8 Statistical analyses

Demographic characteristics of the study population were presented in frequencies and percentages. Linear regression analysis was conducted to determine if demographics and past infection status were associated with higher knowledge score in dengue and dengue vaccination awareness. Chi-squared test was conducted to find association between age group and measures to increase uptake of dengue preventive measures. Statistical significance was set at *p* < 0.05. All analysis was carried out using R3.5.2 with RStudio and tidyverse library.

### 2.9 Ethical approval

The study obtained ethics approval from the institution review board at the center of higher learning.

## 3 Results

### 3.1 Demographic profile of the study population

A total of 1,822 e-questionnaires were distributed via the WhatsApp and Telegram portals, and 624 participants completed the survey (response rate 34.2%). The mean age of participants were 17.4 years ± 1.84 with 59.3% females. Majority of the participants were Chinese (89.9%) and do not have any past dengue infection (95.7%). The characteristics of the participants are shown in Table 1.

**Table 1:** Characteristics and dengue awareness of study participants (*N* = 624).

Characteristics	Total, *n* (%)
Age in years, mean (SD)	17.4 (1.84)
*Gender*	
Female	370 (59.3)
Male	254 (40.7)
*Ethnicity*	
Chinese	561 (89.9)
Malay	25 (4.0)
Indian	28 (4.5)
Others	10 (1.6)
*Past dengue infection*	
No	597 (95.7)
Yes	27 (4.3)
*Dengue awareness*	
Total awareness score (0–20), mean (SD)	14.1 (2.8)
*a. A person can be infected with dengue more than once*	
Strongly agree	259 (41.5)
Agree	266 (42.6)
Neutral	89 (14.3)
Disagree	8 (1.3)
Strongly disagree	2 (0.3)
*b. Dengue infection can be fatal*	
Strongly agree	348 (55.8)
Agree	225 (36.1)
Neutral	39 (6.2)
Disagree	11 (1.8)
Strongly disagree	1 (0.2)
*c. Dengue vaccine is available to local people*	
Strongly agree	90 (14.4)
Agree	119 (19.1)
Neutral	344 (55.1)
Disagree	57 (9.1)
Strongly disagree	14 (2.2)
*d. Dengue vaccine can be administered for selected people aged 12–45 years*	
Strongly agree	76 (12.2)
Agree	146 (23.4)
Neutral	374 (59.9)
Disagree	20 (3.2)
Strongly disagree	8 (1.3)
*e. Dengue vaccine is effective in reducing the infection*	
Strongly agree	98 (15.7)
Agree	278 (44.6)
Neutral	212 (34.0)
Disagree	31 (5.0)
Strongly disagree	5 (0.8)

### 3.2 Knowledge score of dengue and dengue vaccination

Table 1 also shows the factors associated with participant’s dengue knowledge score. Overall, the mean dengue knowledge scores of participants were 14.1 ± 2.8. Majority of participants had awareness of ability to contract dengue more than once, dengue infection can be fatal and dengue vaccine effectiveness in reducing infection. However, most participants were not aware of availability and age group criteria of dengue vaccination. Univariate analysis conducted show that teenagers (15–19 years old) was significantly associated with higher knowledge score (β = 0.82, 95%CI = 0.13–1.51, *p* = 0.021). After adjusting for gender, ethnicity and past infection status, age remained significant (β = 0.9, 95%CI = 0.19–1.61, *p* = 0.013). Gender, ethnicity, and past infection status were not significant to predict knowledge scores (Table 2).

**Table 2:** Linear regression on factors associated with dengue knowledge scores.

Variables	Unadjusted coefficient β (95%CI)	*P*-value	Adjusted coefficient β (95%CI)	*P*-value
*Age (years)*				
15–19	0.82 (0.13–1.51)	0.021	0.9 (0.19–1.61)	0.013
20–24	Ref	–	Ref	–
*Gender*				
Female	0.03 (−0.42–0.48)	0.888	0.02 (−0.44–0.47)	0.943
Male	Ref	–	Ref	–
*Ethnicity*				
Chinese	−0.19 (−1.95–1.57)	0.831	−0.1 (−1.86–1.67)	0.915
Malay	0.62 (−1.45–2.69)	0.556	0.84 (−1.23–2.92)	0.425
Indian	−0.09 (−2.12–1.95)	0.934	0.25 (−1.81–2.3)	0.814
Others	Ref	–	Ref	–
*Past dengue infection*				
No	−0.04 (−1.12–1.05)	0.946	−0.04 (−1.13–1.04)	0.941
Yes	Ref	–	Ref	–

### 3.3 Awareness and perception of effectiveness of prevention measures

**Figure 1:**
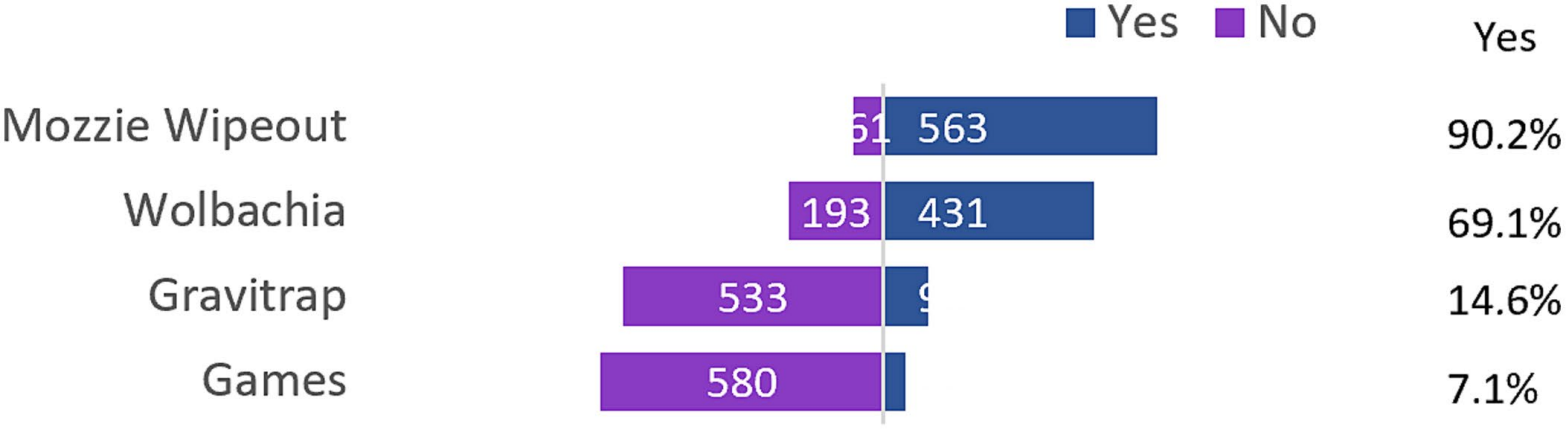
Awareness of dengue prevention measures.

**Figure 2:**
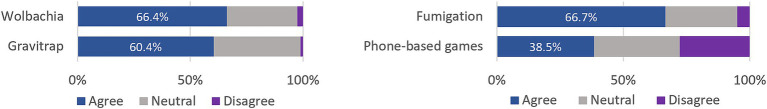
Effectiveness of vector control measures.

Most participants were aware of the Mozzie Wipeout campaign (90.2%) followed by the release of Wolbachia mosquitos (69.1%). However, majority of participants (92.9%) were not aware of games that raises awareness in vector control and the Gravitrap device (85.4%) (Figure 1). Among youths that were aware of Wolbachia and Gravitrap, approximately two-thirds of them felt that it was effective in reducing dengue infection rates. About 4 in 10 also agreed that phone-based games would be effective in raising awareness of dengue and its preventive measures (Figure 2).

### 3.4 Perceived effectiveness of dengue prevention measures

**Figure 3:**
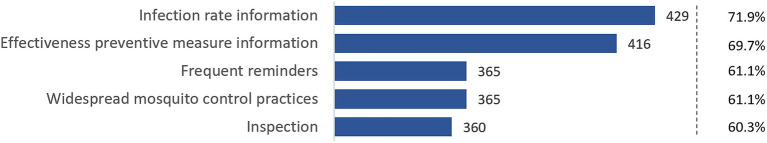
Receptivity toward dengue preventive measures.

Among the 5 proposed measures that will aid in improving uptake of dengue preventive measures, participants listed information about the current dengue infection rate (71.9%) to be most effective, followed by information on the effectiveness of preventive measures (69.6%). The remaining measures such as frequent reminders, inspection by authorities and widespread mosquito control practices were least popular (~60%) (Figure 3).

Subgroup analysis showed that teenagers (15–19 years) were more likely to uptake dengue preventive measures if there were widespread mosquito control practices adopted compared to young adults (20–24 years) (69.1% vs. 42.3%, *p* < 0.001). Other measures to encourage uptake of dengue preventive measures were not statistically significant as shown in Table 3.

**Table 3:** Measures to increase uptake of preventive measures among adolescents (*N* = 624).

Characteristics	Total, *n* (%)	15–19 years old, *n* (%)	20–24 years old, *n* (%)	*P*-value
Total	624 (100)	553 (88.6)	71 (11.4)	
*Inspections*				0.517
No	246 (39.4)	215 (38.9)	31 (43.7)	
Yes	378 (60.6)	338 (61.1)	40 (56.3)	
*Infection rate information*				0.173
No	181 (29.0)	155 (28.0)	26 (36.6)	
Yes	443 (71.0)	398 (72.0)	45 (63.4)	
*Effectiveness preventive measure information*				0.059
No	190 (30.4)	161 (29.1)	29 (40.8)	
Yes	434 (69.6)	392 (70.9)	42 (59.2)	
*Widespread mosquito control practices*				< 0.001
No	212 (34.0)	171 (30.9)	41 (57.7)	
Yes	412 (66.0)	382 (69.1)	30 (42.3)	
*Frequent reminders*				0.872
No	245 (39.3)	216 (39.1)	29 (40.8)	
Yes	379 (60.7)	337 (60.9)	42 (59.2)	

## 4 Discussion

Majority of the youths in this study population possess good knowledge of dengue infection. 84.1% of participants are aware that dengue infection can be fatal and 91.9% of them agree that a person can be infected with dengue more than once. This is consistent with another study by Mustafa et al. on knowledge, attitude and preventive behavior regarding dengue fever among youth in neighboring Malaysia (18). Such finding is attributed to the high literacy rate among the youths in these dengue endemic countries in which reports of its outbreaks frequently appear in various media (19).

However, only 33.5% of the participants are aware of the availability of dengue vaccine in Singapore, and a similar percentage are aware of the age eligibility criteria for such vaccination. The comparatively low awareness of dengue vaccine can be attributed to the lack of publicity by the authorities because of its limited use in only previously infected individuals who are aged 12 to 45 years old. Currently the transmission of information is mainly channeled from general practitioners (GPs) or clinicians to patients. A comprehensive program to educate and increase public awareness of dengue vaccine and its use is necessary to motivate individuals who meet the eligibility criteria to proactively get vaccinated. Such a proactive approach will help to reduce the risk of future dengue infection within the community. Other ways to enhance uptake of dengue vaccine among suitable individuals can be more extensively addressed in a more detailed questionnaire designed to explore the reasons for poor uptake of dengue vaccine among the local youths.

Teenagers (15–19 years old) tend to have higher knowledge scores in dengue disease and its vaccine. The higher knowledge scores may be related to close collaboration between different government agencies and schools in Singapore in promoting awareness of dengue in recent years. NEA works with schools to organize anti-dengue outreach programs (20). The importance of such educational programs is further emphasized by the ongoing global warming projected to lead to a record number of dengue infection and schools being potential dengue clusters (3, 21). Similar findings on increased awareness of students are observed in a few Asia-Pacific countries after an intervention program (22–24). Raising the dengue literacy of youths through schools can potentially spread their knowledge and practice to other family and household members.

In this study, dengue infection status is not a significant factor to predict knowledge level, with dengue knowledge scores of previously infected participants (14.2 ± 3.45), which are similar to those who are naïve to the infectious disease (14.1 ± 2.78). Studies in Aceh in Indonesia and Kachanadit district in Southern Thailand also reported that participant’s previous dengue infection status was not associated with their dengue knowledge (25, 26). Interestingly, the result identifies a missed opportunity to vaccinate those who were infected, which will reduce their risks of future dengue infection. GPs or clinicians can also share vector control measures with these infected youths during their consultation, leveraging on the well-established primary care network in Singapore. Local primary healthcare clinics are easily accessible to residents, maximizing efforts by healthcare professionals to raise the awareness of the public, including the youth on dengue prevention measures.

Most study participants were aware of the dengue prevention initiatives by NEA in controlling the spread of dengue. Approximately 90 and 69% of participants were aware of the Mozzie Wipeout campaign and the use of Wolbachia mosquitoes, respectively. Participant’s knowledge of such initiatives is likely due to the guides and toolkits distributed to each household on such measures. Nevertheless, high level of awareness of these measures may not necessarily translated to the relevant actions to curb mosquito breeding. An article by Ong et al. reported knowledge-practice gap in Singapore where motivation plays a role in translating knowledge into practice of preventive measures (27). Similar inertia was observed among the local population in Malaysia and Northern Thailand (28, 29). The motivation to initiate preventive measures was largely dependent on the health beliefs of a person. In Malaysia, a focus group discussion on health beliefs relating to dengue prevention identified low self-efficacy, low perceived susceptibility, and perceived lack of benefit as barriers to the uptake of dengue preventive measures (30). Further research to develop and test multi-dimensional interventions which can effectively address these barriers is needed to improve dengue prevention measures. In addition, practice of dengue prevention is dependent on community efforts. Other members of the community besides the youths also play a part in dengue prevention. The study starts by looking at the youths, the group that has the second highest incidence of dengue in the community as they can be engaged to further improve dengue prevention because of their receptivity and ability to adopt new behaviors. There was no preceding studies on this area among youths in Asia thus the findings of this study will be useful to enhance vector control and make a difference in reducing incidence of dengue in the local area.

Approximately 40% of participants in this study agreed that phone-based games would be effective in raising awareness of dengue infection and its vaccine although only 7.1% of participants were aware of it being used as a vector control measure. The positive perception of phone-based serious games as an effective educational tool raises its potential for application among the younger gamers and complement current modality such as talks in school to improve their dengue literacy. Usage of games for educational purposes on dengue and its prevention has gained traction in the past decade, progressing from computer games to serious games (31). A recent study conducted by Tan et al. reported that the serious game “Sam’s Mozzie Adventure” improved public awareness of dengue preventive measures (32). The local setting is conducive to implement serious games, especially almost all of youths in Singapore are equipped with a smartphone and 82% of them engage in online games using their phones (33, 34). Hence, serious games could potentially play a crucial role in activating them to take on dengue preventive measures.

Information on dengue infection rate and the effectiveness of specific preventive measures are the two main important factors to increase the youth’s uptake of dengue preventive measures. Comparison across age groups revealed significant difference between teenagers (69.1%) and young adults (42.3%) in undertaking dengue preventive measures. Educational efforts on dengue prevention should be sustained among the youths as they mature via regular and targeted community health publicity and engagement programs.

### 4.1 Limitations

The cross-sectional study design allows only associations between the stipulated factors and awareness level of dengue prevention measures. The study population comprised largely of students of public secondary and tertiary institutions due to the study team’s personal contacts in their social media portals, and those from private schools or who had left school are likely to be excluded.

Interviewer bias was minimized based on the electronic self-administered survey. However, selection bias potentially remains, as those who are health-conscious, concerned or knowledgeable about dengue were more willing to become participants. Thus, the results cannot be generalized to the entire local youth population. Future studies can be conducted to extend the same questionnaire to the groups that were not well represented in this study to have a more comprehensive understanding of the youths on dengue and its prevention.

### 4.2 Strengths

Globally studies targeting youths on dengue infection and vector control measures are relatively sparse. The study offers a quick, inexpensive way through social media platforms to reach out to youth participants from diverse background.

## 5 Conclusion

The surveyed population especially the teenagers aged 15–19 years old has good knowledge of dengue, but awareness of its vaccine is low. While encouraging vaccination among the at-risk population, vector control measures are essential in controlling the spread of the endemic disease. It can be a springboard for further studies to identify the essential steps to translate knowledge to appropriate mitigating actions. More efforts may need to be made to engage the young adults (20–24 years old) to take proactive steps to prevent dengue. Social and digital media can be used to reach out to the youths as many have access to these portals and are savvy in the use of technology. They can assist to disseminate information on dengue disease, neighborhood infection rates, vaccination, and preventive measures, in tandem with the involvement of schools and GPs as a proactive, multifaceted community preparedness program to strengthen vector control measures against dengue and other mosquito-borne diseases.

## Data availability statement

The original contributions presented in the study are included in the article/supplementary material, further inquiries can be directed to the corresponding author.

## Author contributions

WS: Writing – review & editing, Writing – original draft. KG: Writing – review & editing, Writing – original draft. DN: Writing – review & editing, Writing – original draft. KK: Writing – review & editing, Writing – original draft. NT: Writing – review & editing.
